# Acute Angina Following the Use of Salol

**Published:** 1892-07

**Authors:** 


ACUTE ANGINA FOLLOWING THE USE OF SALOL.—Morel-Lavallee records the case of a young woman, a syphilitic, who, having otorrhœa, was treated by insufflations of salol. Three days after, fever appeared, with enormous swelling of the external auditory canal, the isthmus of the fauces, and of the uvula. In three or four days these symptoms disappeared. The cause of the trouble was, according to the author, the decomposition of the salol into its two component parts, which was brought about by the presence of a fatty substance.—*Archives Internationales de Laryngologie*, 1891, p. 140

